# ﻿Four new freshwater crab species of the genus *Megapleonum* Huang, Shih & Ahyong, 2018 (Crustacea, Decapoda, Potamidae) from Guangdong, China

**DOI:** 10.3897/zookeys.1244.148112

**Published:** 2025-07-02

**Authors:** Chao Huang, Hsi-Te Shih, Shane T. Ahyong

**Affiliations:** 1 Australian Museum, 1 William St, Sydney NSW 2010, Australia Australian Museum Sydney Australia; 2 Department of Life Science and Global Change Biology Research Center, National Chung Hsing University, Taichung 402, Taiwan National Chung Hsing University Taichung Taiwan; 3 School of Biological, Earth and Environmental Sciences, University of New South Wales, Kensington, NSW 2052, Australia University of New South Wales Kensington Australia

**Keywords:** 16S rDNA, aquatic species, China, new taxa, systematics

## Abstract

Four new species of the poorly known genus *Megapleonum* Huang, Shih & Ahyong, 2018, are described from Guangdong Province, China: *Megapleonumfalx***sp. nov.** from Huizhou City, *M.yangdongense***sp. nov.** from Yangjiang City, and both *M.ferrumequinum***sp. nov.** and *M.wangjiani***sp. nov.** from Maoming City. These four new species are all morphologically and genetically distinct from each other and the two known congeners *Megapleonumehuangzhang* Huang, Shih & Ahyong, 2018 and *Megapleonumshenzhen* Huang & Mao, 2021. The extremely divergent morphology of the gonopod 1 of these species alone immediately sets them apart, but there are also distinct differences in other characters, including the carapace, ambulatory leg, and maxilliped 3 exopod flagellum. A phylogeny constructed using the mitochondrial 16S rDNA reveals that all species of *Megapleonum* form a deep-rooted monophyletic group with significant interspecific genetic distances, supporting the generic placement and specific treatments of these new taxa. A key to the species of *Megapleonum* is also provided.

## ﻿Introduction

Located in the Huanan freshwater zoogeographic province, the Chinese province of Guangdong has a high diversity of freshwater crabs yet remains relatively poorly sampled (Huang et al. 2020; Mao and Huang 2020). The Guangdong endemic genus *Megapleonum* Huang, Shih & Ahyong, 2018, was established to accommodate the type species *Megapleonumehuangzhang* Huang, Shih & Ahyong, 2018, which was discovered from Yangxi County, Yangjiang. This genus is immediately recognizable by its wide male abdomen, a character not seen in other East Asian potamids (Huang et al. 2018). *Megapleonumshenzhen* Huang & Mao, 2021, was described from Shenzhen City a few years later, becoming the second species reported in the previously monotypic genus. However, the large geographical separation between the localities of these two narrow-range species suggests the likely discovery of more species of this genus in the area between. Zoological surveys during the past few years have yielded four new species of *Megapleonum*, formally described herein.

## ﻿Materials and methods

Specimens were collected by hand, preserved in 75–95% ethanol, and deposited in the collections of the Sun Yat-sen Museum of Biology, Sun Yat-sen University, Guangzhou, China (**SYSBM**), the Museum of Aquatic Organism, Institute of Hydrobiology, Chinese Academy of Sciences, Wuhan, China (**IHB**), Zoological Collections of the Department of Life Science, National Chung Hsing University, Taichung, Taiwan (**NCHUZOOL**), and the Jiangsu Key Laboratory for Biodiversity and Biotechnology, College of Life Sciences, Nanjing Normal University, Nanjing, China (**NNU**). The terminology used primarily follows that of Dai (1999) and Davie et al. (2015). Carapace length (**CL**) is measured along the dorsal midline. Carapace width (**CW**) is the greatest width, measured across the branchial margins. The male gonopods 1 and 2 are abbreviated as **G1** and **G2**, respectively. Measurements (mm) are of the carapace width and length, respectively.

Sequences of the mitochondrial 16S rDNA were obtained from specimens using the universal DNA purification kit (Tiangen, Beijing, China). A region of ~550 base pairs (bp) of the 5’-end of the 16S gene was selected for amplification with polymerase chain reaction (PCR) using the primers 1471 and 1472 (Crandall and Fitzpatrick 1996). The PCR conditions for the above primers were an initial denaturation for 3 minutes at 94 °C, followed by 35 cycles of denaturation for 30 s at 94 °C, annealing for 45 s at 45 °C, and extension for 60 s at 72 °C, followed by a final extension for 3 minutes at 72 °C. Sequences were obtained by automated sequencing (Applied Biosystems), and have been deposited in NCBI GenBank under the accession number PQ776773–PQ776781. According to Huang et al. (2018), the genus *Megapleonum* belongs to the “China-East Asia Islands” clade, supported by Shih et al. (2009) and Pan et al. (2022), so the tree was rooted using the closely related “China” clade (Shih et al. 2009).

Sequences of 16S were aligned with the aid of the MUSCLE function of MEGA v. 11 (Tamura et al. 2021). The variable regions in loop regions of the 16S that could not be aligned adequately were excluded (Shih et al. 2009; Huang et al. 2018), and the obtained 503 bp segment were used in the phylogenetic analyses. The best-fitting model for sequence evolution was determined by PartitionFinder v. 2.1.1 (Lanfear et al. 2017) and selected based on the Bayesian information criterion (BIC). The obtained best model (GTR+I+G) was subsequently employed for Bayesian inference (BI) and maximum likelihood (ML) analyses. BI analysis was performed with MrBayes v. 3.2.3 (Ronquist et al. 2012), running four chains for 10 million generations across four independent runs, with trees sampled every 1,000 generations. The convergence of chains was determined using the average standard deviation of split frequency values, which remained below the recommended threshold of 0.01 (Ronquist et al. 2020). Accordingly, the first 700 trees were discarded as “burn-in”. ML analysis was performed using IQ-TREE v. 2.2.0 (Minh et al. 2020) with the best model, and 30,000 ultrafast bootstrap replicates were generated (Hoang et al. 2017). Bp differences and pairwise estimates of Kimura 2-parameter (K2P) distances (Kimura 1980) for genetic diversities between specimens were calculated with MEGA.

## ﻿Taxonomy

**Family Potamidae Ortmann, 1896**

**Subfamily Potamiscinae Bott, 1970**

### ﻿ Megapleonum

Taxon classificationAnimaliaDecapodaPotamidae

Huang, Shih & Ahyong, 2018

5B3B1E37-FE7C-52C9-956D-D1C067AFCC3A

#### Diagnosis.

Carapace broader than long, dorsal surface slightly convex; postorbital, epigastric cristae confluent or almost confluent; external orbital angle bluntly triangular, confluent or almost confluent with anterolateral margin. Epistome buccal margin with triangular median lobe. Maxilliped 3 ischium longitudinally subquadrate, length ~0.7× width; exopod reaching slightly beyond distal edge of ischium, flagellum absent to well-developed. Male anterior thoracic sternum very broad, width 1.8–2.0× length. Male pleon large, broadly triangular to sublinguiform, tip of telson rounded. Female pleon linguiform to subovate. G1 large, sinuous, reaching beyond male pleonal locking tubercle, subterminal segment outer margin slightly to strongly concave, terminal segment highly variable. G2 subterminal segment tapering, bent to slightly bent outwards distally, thin to thick flagelliform terminal segment. Vulvae ovate, large, reaching suture of sternites 5/6, relatively widely separated.

#### Composition.

*Megapleonumehuangzhang* Huang, Shih & Ahyong, 2018 (type species), *Megapleonumfalx* sp. nov., *M.ferrumequinum* sp. nov., *Megapleonumshenzhen* Huang & Mao, 2021, *M.yangdongense* sp. nov., and *M.wangjiani* sp. nov.

#### Remarks.

With the description of four new species herein, the diagnosis of the genus has to be revised. The significant variation within the genus, particularly in the form of the G1 poses challenges to establishing a uniform diagnosis. However, the key distinguishing features include the confluent or almost confluent postorbital and epigastric cristae, and external orbital angle and anterolateral margin, very broad male anterior thoracic sternum, large male pleon and the ovate, large vulvae, which extend to the suture of sternites 5/6 and are relatively widely spaced.

### ﻿Key to the species of *Megapleonum*

**Table:** 

1	Ambulatory legs with dense long setae	**2**
–	Ambulatory legs without dense long setae	**4**
2	Carapace lateral margin with dense long setae	**3**
–	Carapace lateral margin without dense long setae	***Megapleonumwangjiani* sp. nov.**
3	Maxilliped 3 exopod with flagellum	***Megapleonumfalx* sp. nov.**
–	Maxilliped 3 exopod without flagellum	***Megapleonumferrumequinum* sp. nov.**
4	Maxilliped 3 exopod with short but distinct flagellum (as long as dactylus)	***Megapleonumyangdongense* sp. nov.**
–	Maxilliped 3 exopod flagellum absent or vestigial bud	**5**
5	G1 apex terminating in single lobe	**6**
–	G1 apex terminating in two lobes	***Megapleonumshenzhen***
6	G1 apex lobe folded	***Megapleonumehuangzhang***
–	G1 apex lobe not folded	***Megapleonum* sp. “Taishan**”

### ﻿ Megapleonum falx sp. nov.

Taxon classificationAnimaliaDecapodaPotamidae

FEBCE5AD-8B23-5E49-A600-56E5B8027C78

https://zoobank.org/8F1A2D8E-5CC3-4DA2-AF9D-50DC0C1885D7

Figs 1 , 2

#### Type material.

***Holotype***: • SYSBM 002142, male (19.6 × 15.8 mm), Baima Village, Huidong County, Huizhou City, Guangdong Province, China, 23.03°N, 115.07°E, hill stream under rock, coll. Song-Bo Wang, August 2019.

#### Diagnosis.

**Figure 1.**
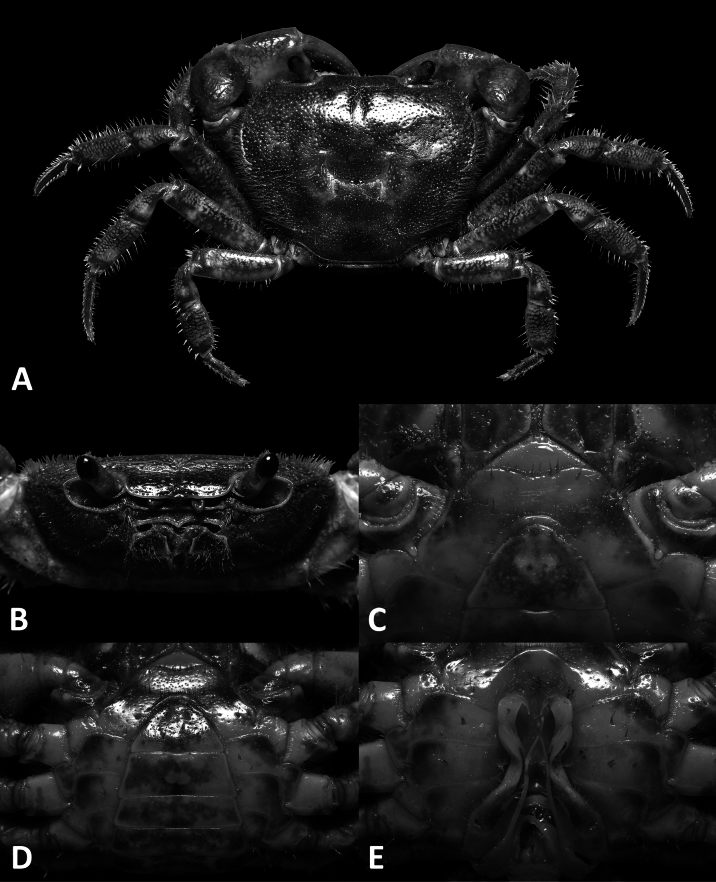
*Megapleonumfalx* sp. nov., male holotype (19.6 × 15.8 mm), SYSBM 002142. Dorsal habitus (**A**); cephalothorax, anterior view (**B**); anterior thoracic sternum (**C**); anterior thoracic sternum and pleon, ventral view (**D**); sterno-pleonal cavity with G1 in situ, ventral view (**E**).

**Figure 2.**
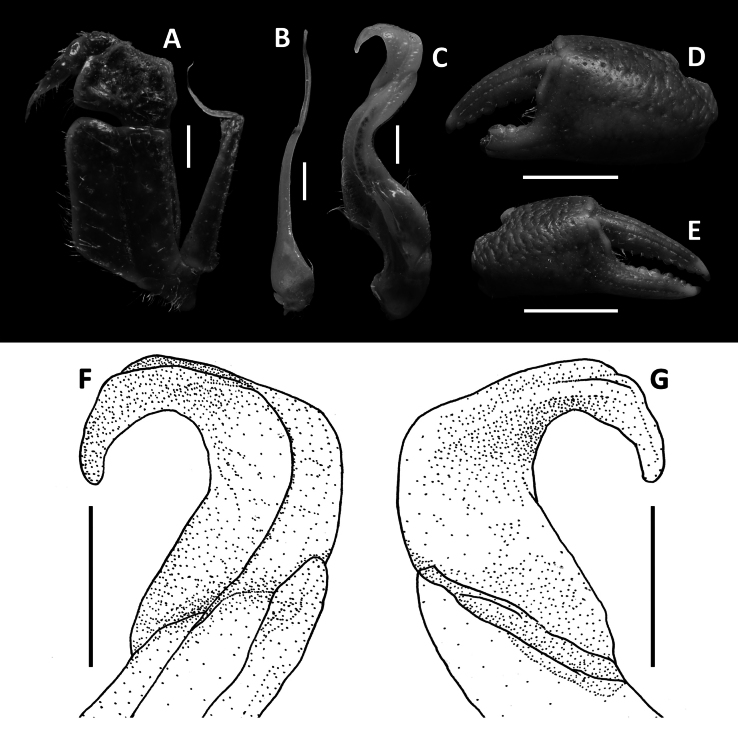
*Megapleonumfalx* sp. nov., male holotype (19.6 × 15.8 mm), SYSBM 002142. Left maxilliped 3 (**A**); left G2, pleonal view (**B**); left G1, ventral view (**C**); major cheliped, partially damaged (**D**); minor cheliped (**E**); left G1 terminal segment, ventral view (**F**); left G1 terminal segment, dorsal view (**G**). Scale bars: 1.0 mm (**A**–**C**, **F**, **G)**, 5.0 mm **(D**, **E)**.

Carapace broader than long, dorsal surface slightly convex, lateral margins covered in dense setae; postorbital, epigastric cristae confluent (Fig. 1A). Maxilliped 3 sparsely covered in long setae; merus width ~1.4× length; ischium width ~0.7× length; exopod reaching beyond anterior edge of ischium, flagellum as long as merus width (Fig. 2A). Ambulatory legs with dense setae; pereiopod 5 dactylus ~as long as propodus (Fig. 1A). Male anterior thoracic sternum very broad, width ~1.8× length (Fig. 1C). Male pleon large, broadly triangular, pleonite 6 width ~2.6× length, telson width ~1.8× length (Fig. 1D). G1 large, strongly sinuous, tip exceeding suture between thoracic sternites 4/5 in situ (Fig. 1E); subterminal segment length ~3.2× length of terminal segment (Fig. 2C). Subterminal segment outer margin strongly concave; terminal segment strongly curved inwards, strongly tapering, sickle shaped, tip pointed downwards in dissected view (Fig. 2C, F, G). G2 subterminal segment tapering, bent outwards distally, flagelliform terminal segment ~1.8× length of subterminal segment, apex blunt (Fig. 2B).

#### Description of male holotype.

Carapace broader than long, ~1.2× as wide as long; regions not pronounced, dorsal surface slightly convex, finely pitted; dense setae along lateral margins (Fig. 1A). Frontal margin almost straight, deflexed (Fig. 1A, B). Epigastric cristae and postorbital cristae relatively smooth, confluent; bifurcated groove between epigastric cristae (Figs 1A, 2A). Branchial regions not swollen (Fig. 1A, B). Cervical groove shallow but visible (Fig. 1A). Mesogastric region flat (Fig. 1A). External orbital angle broadly triangular, outer margin slightly convex, confluent with anterolateral margin (Fig. 1A, B). Epibranchial tooth granular, indistinct (Fig. 1A, B). Anterolateral margin lined with indistinct single or partially fused granules; posterolateral margin posteriorly convergent (Fig. 1A); posterolateral surface slightly rugose (Fig. 1A). Orbits regular; supraorbital margins weakly cristate, infraorbital margins lined with fused granules (Fig. 1B). Eyes normal (Fig. 1A, B). Sub-orbital, pterygostomial and sub-hepatic regions generally smooth, pitted (Fig. 1B). Antennules large, folded within broad fossae; antennae very short (Fig. 1B). Median lobe of epistome buccal margin broadly triangular, lateral margins slightly sinuous (Fig. 1B).

Maxilliped 3 sparsely covered in long setae, merus subtrapezoidal, with slight median depression, width ~1.4× length; ischium subtrapezoidal with shallow median sulcus, distomesial margin rounded, width ~0.7× length. Exopod reaching proximal one-third of merus; flagellum as long as merus width (Fig. 2A).

Chelipeds (pereiopod 1) subequal (Fig. 2D, E). Merus trigonal in cross-section, surfaces generally smooth; outer dorsal and ventral margins slightly crenulated, inner margin lined with large granules (Fig. 1A, B). Carpus dorsal surface slightly rugose, with large spike at inner-distal angle, spinule at base (Fig. 1A). Major cheliped palm length ~1.5× height; dactylus 0.7× palm length (Fig. 2D, E). Palm surface pitted, occlusal margin of fingers with 7–9 irregular blunt teeth, with small gape when closed (Fig. 2D, E).

Ambulatory legs (pereiopods 2–5) slender, covered with setae (Fig. 1A). Pereiopod 3 merus 0.6× CL (Fig. 1A). Pereiopod 5 propodus length 1.7× height, approximately as long as dactylus (Fig. 1A).

Male thoracic sternum generally smooth, pitted, setae at margins and sparsely on sternites; sternites 1–4 width ~1.8× length; sternites 1, 2 fused to form broad triangle; fused sternites 1, 2 demarcated from sternite 3 by sinuous transverse sulcus, sulcus lined with setae; sternites 3, 4 fused without obvious demarcation other than a line of setae (Fig. 1C). Male sterno-pleonal cavity reaching anteriorly slightly beyond mid-length of cheliped coxa (Fig. 1C). Male pleonal locking tubercle positioned at mid-length of sternite 5 (Fig. 1E).

Male pleon large, broadly triangular; somites 3–6 progressively narrower, not entirely confluent with each other; somite 6 width approximately 2.6× length; telson width 1.8× length; lateral margins almost straight, apex rounded (Fig. 1D).

G1 large, strongly sinuous, tip exceeding suture between thoracic sternites 4/5 in situ (Fig. 1E); subterminal segment length ~3.2× length of terminal segment (Fig. 2C). Subterminal segment outer margin strongly concave; terminal segment strongly curved inwards, strongly tapering, sickle shaped, tip pointed downwards in dissected view (Fig. 2C, F, G). G2 subterminal segment tapering, bent outwards distally, flagelliform terminal segment ~1.8× length of subterminal segment, apex blunt (Fig. 2B).

#### Colour in life.

Generally camouflage-brown all over.

#### Habitat.

Unknown. The only specimen collected was found in the lower reaches of the hill stream, but further collection efforts in the same area and further upstream yielded no crabs at all. We consider it likely that the primary habitat of this species is higher up the mountain, which reaches above 1000 m above sea level, and that this lone specimen that was collected was washed downstream in a flooding event.

#### Distribution.

Baima Village, Huidong County, Huizhou City, Guangdong Province, China.

#### Etymology.

The species name is the Latin word *falx* which means sickle-shaped. It alludes to the sickle-shaped G1 terminal segment of this species.

#### Remarks.

**Figure 3.**
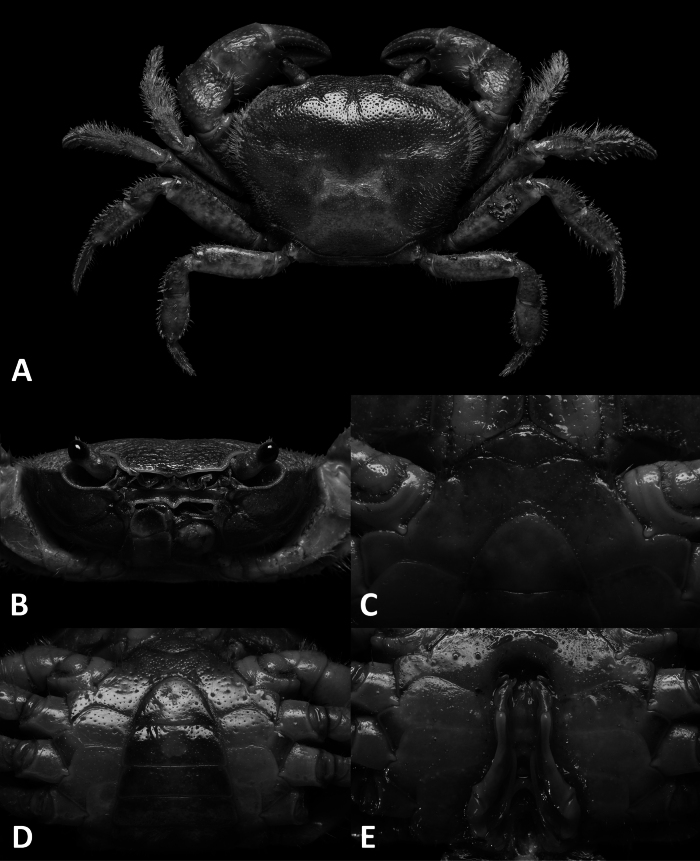
*Megapleonumferrumequinum* sp. nov., male holotype (16.6 × 13.1 mm), SYSBM 002143. Dorsal habitus (**A**); cephalothorax, anterior view (**B**); anterior thoracic sternum (**C**); anterior thoracic sternum and pleon, ventral view (**D**); sterno-pleonal cavity with G1 in situ, ventral view (**E**).

**Figure 4.**
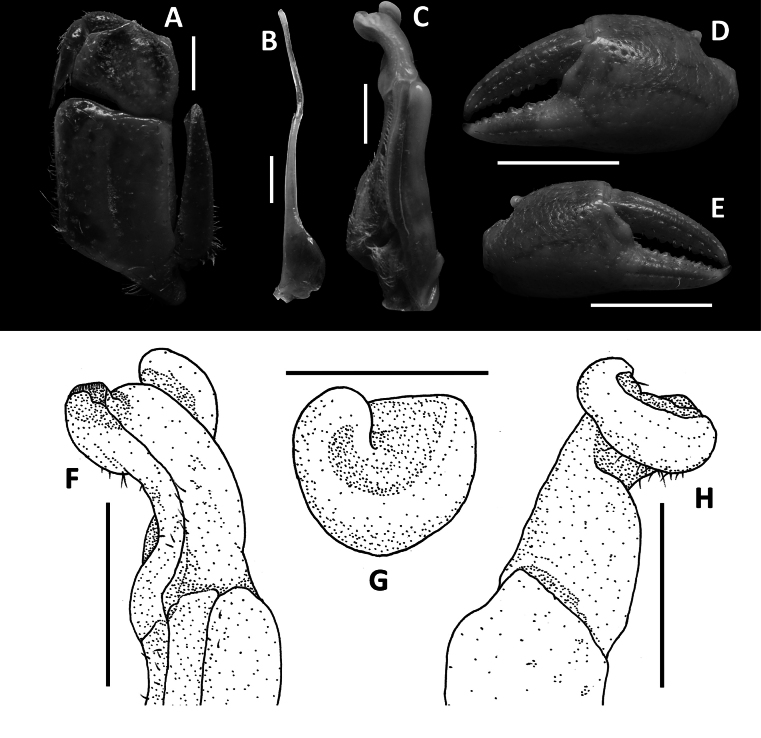
*Megapleonumferrumequinum* sp. nov., male holotype (16.6 × 13.1 mm), SYSBM 002143. Left maxilliped 3 (**A**); left G2, pleonal view (**B**); left G1, ventral view (**C**); major cheliped (**D**); minor cheliped (**E**); left G1 terminal segment, ventral view (**F**); left G1 horse-shoe structure viewed from the top (**G**); left G1 terminal segment, dorsal view (**H**). Scale bars: 1.0 mm **(A**–**C**, **F**–**H)**, 5.0 mm **(D**, **E)**.

The wide male anterior thoracic sternum, large and broadly triangular male abdomen and large and sinuous G1 of *Megapleonumfalx* sp. nov. fit the diagnosis of the genus. In possessing thick setae on the carapace margins and ambulatory legs, *M.falx* sp. nov. is most similar to *M.ferrumequinum* sp. nov. The sickle-shaped G1 terminal segment of *M.falx* sp. nov., however, is unlike any other congener and immediately distinguishes it (Fig. 2C, F, G). Apart from the G1, *M.falx* sp. nov. also has a flatter carapace dorsal surface when compared to *M.ferrumequinum* sp. nov. (Figs 1B, 3B). The flagellum of the maxilliped 3 exopod is present in *M.falx* sp. nov. whereas it is absent in *M.ferrumequinum* sp. nov. (Fig. 2A vs Fig. 4A). The male abdomen is also obviously wider in *M.falx* sp. nov., with the somite 6 width approximately 2.6× length (Fig. 1D) (vs narrower in *M.ferrumequinum* sp. nov., sixth somite width approximately 2.5× length, Fig. 3D). The relative length of the pereiopod dactylus to the propodus is also different, being approximately the same length in *M.falx* sp. nov. and in *M.ferrumequinum* sp. nov., having a shorter dactylus (Figs 1A, 3A). More detailed comparisons can be found in Table 1.

**Table 1.** Morphological comparisons between the species within the genus *Megapleonum*.

Character	*Megapleonumfalx* sp. nov.	*Megapleonumferrumequinum* sp. nov.	*Megapleonumwangjiani* sp. nov.	*Megapleonumyangdongense* sp. nov.	*Megapleonumehuangzhang*	*Megapleonumshenzhen*
Carapace	Dense long setae at lateral margins, epigastric cristae and postorbital cristae confluent (Fig. 1A)	Dense long setae at lateral margins, epigastric cristae and postorbital cristae almost confluent (Fig. 3A)	Sparse short setae at lateral margins, epigastric cristae and postorbital cristae almost confluent (Fig. 5)	No setae at lateral margins, epigastric cristae and postorbital cristae confluent (Fig. 8)	No setae at lateral margins, epigastric cristae and postorbital cristae confluent (Huang et al. 2018: fig. 2)	No setae at lateral margins, epigastric cristae and postorbital cristae confluent (Huang and Mao 2021: fig. 1)
Maxilliped 3 exopod	With long flagellum (Fig. 2A)	With no flagellum (Fig. 4A)	With no flagellum (Fig. 7A)	With short flagellum (Fig. 10A)	With no flagellum (Huang et al. 2018: fig. 3D)	With vestigial flagellum (Huang and Mao 2021: fig. 3A)
Ambulatory legs	With dense long setae (Fig. 1A)	With dense long setae, particularly pereiopods 2 and 3 (Fig. 3A)	With dense long setae, particularly pereiopods 2 and 3 (Fig. 5)	With short setae (Fig. 8)	With short setae (Huang et al. 2018: fig. 2)	With short setae (Huang and Mao 2021: fig. 1)
Male anterior thoracic sternum width length ratio	1.8	1.9	2.0	1.9	1.9 (Huang et al. 2018)	1.9 (Huang and Mao 2021)
Male pleonite 6 width to length ratio	2.6	2.5	2.6	2.4	2.6 (Huang et al. 2018)	2.6 (Huang and Mao 2021)
Male telson width to length ratio	1.8	1.7	1.8	1.8	1.7 (Huang et al. 2018)	2.0 (Huang and Mao 2021)
G1 terminal segment	Strongly tapering, sickle shaped (Fig. 2F, G)	Long and curved with horseshoe shaped structure (Fig. 4F–H)	Short, rounded, distal-ventral region with long setae, tip presenting as protrusion on outer margin (Fig. 11A, B)	Short, bifurcated, prong shaped (Fig. 11C, D)	Bent outwards and folded inwards with large flap on ventral side (Huang et al. 2018: fig. 3C)	Stout, goose-head-shaped (Huang and Mao 2021: fig. 4G)
G2 subterminal segment proximal region	Small (Fig. 2B)	Small (Fig. 4B)	Very small (Fig. 7B)	Large (Fig. 10B)	Large (Huang et al. 2018: fig. 3A)	Large (Huang and Mao 2021: fig. 3B)
Female pleon	Ovate	Ovate	Ovate (Fig. 6E)	Ovate (Fig. 9E)	Linguiform (Huang et al. 2018: fig. 4C)	Linguiform (Huang and Mao 2021: fig. 2E)

### ﻿ Megapleonum ferrumequinum sp. nov.

Taxon classificationAnimaliaDecapodaPotamidae

7FB5A7F2-3BF5-5961-9415-E29FCBFBB8C3

https://zoobank.org/3E312295-C1E5-47FD-B90E-086F02A72785

Figs 3 , 4 , 13A

#### Type material.

***Holotype***: • SYSBM 002143, male (16.6 × 13.1 mm), Datianding, Dawuling Nature Reserve, Maoming City, Guangdong Province, China, 22.29°N, 111.22°E, dirt road near the summit at night, coll. Jian Wang, June 2018.

#### Diagnosis.

Carapace broader than long, dorsal surface convex, lateral margins covered in dense setae; postorbital, epigastric cristae weak, almost confluent (Fig. 3A). Maxilliped 3 merus width ~1.3× length; ischium width ~0.7× length; exopod reaching slightly beyond anterior edge of ischium, without flagellum (Fig. 4A). Ambulatory legs densely setose; pereiopod dactylus shorter than propodus (Fig. 3A). Male anterior thoracic sternum very broad, width ~1.9× length (Fig. 3C). Male pleon large, sublinguiform, pleonite 6 width ~2.5× length; telson width ~1.7× length (Fig. 3D). G1 large, slightly sinuous, tip exceeding suture between thoracic sternites 4/5 in situ (Fig. 3E); subterminal segment length ~2.5× length of terminal segment (Fig. 4C). Subterminal segment outer margin slightly concave, outer distal margin bulging; terminal segment curved inwards, tip pointed upwards in dissected view, connected to a large mesoanterior-facing horseshoe shaped structure, large thick proximal pad on dorsal side (Fig. 4C, F, G). G2 subterminal segment tapering, slightly bent outwards distally, flagelliform terminal segment thick, ~1.6× length of subterminal segment, apex blunt (Fig. 4B).

#### Description of male holotype.

Carapace broader than long, ~1.3× as wide as long; regions not pronounced, dorsal surface convex; surface finely pitted, dense setae at lateral margins (Fig. 3A). Frontal margin almost straight, deflexed (Fig. 3A, B). Epigastric cristae and postorbital cristae rugose, low, almost confluent; bifurcated shallow groove between epigastric cristae (Fig. 3A). Branchial regions not swollen (Fig. 3A, B). Cervical groove shallow, barely visible (Fig. 3A). Mesogastric region flat (Fig. 3A). External orbital angle broadly triangular, outer margin slightly convex, confluent with anterolateral margin (Fig. 3A, B). Epibranchial tooth granular, indistinct (Fig. 3A, B). Anterolateral margin lined with indistinct single or partially fused granules; posterolateral margin posteriorly convergent (Fig. 3A); posterolateral surface smooth (Fig. 3A). Orbits regular; supraorbital margins weakly cristate, infraorbital margins lined with fused granules (Fig. 3B). Eyes normal (Fig. 3A, B). Sub-orbital, pterygostomial and sub-hepatic regions generally smooth, pitted (Fig. 1B). Antennules large, folded within broad fossae; antennae very short (Fig. 3B). Median lobe of epistome buccal margin broadly triangular, lateral margins straight (Fig. 3B).

Maxilliped 3 merus subtrapezoidal, with slight median depression, width ~1.4× length; ischium subtrapezoidal with shallow median sulcus, distomesial margin rounded, width ~0.7× length. Exopod reaching proximal one-third of merus; flagellum absent (Fig. 4A).

Chelipeds (pereiopod 1) subequal (Fig. 4D, E). Merus trigonal in cross section, surfaces generally smooth, margins lined with long setae; outer dorsal margin slightly crenulated margins slightly crenulated, inner and ventral margin lined with large granules (Fig. 3A, B). Carpus dorsal surface slightly rugose, with large spike at inner-distal angle, spinule at base (Fig. 3A). Major cheliped palm length ~1.5× height; dactylus 0.7× palm length (Fig. 2D, E). Palm surface pitted, occlusal margin of fingers with 7–9 irregular blunt teeth, with small gape when closed (Fig. 2D, E).

Ambulatory legs (pereiopods 2–5) covered with setae, especially dense on pereiopods 2–3 (Fig. 3A). Pereiopod 3 merus 0.6× CL (Fig. 3A). Pereiopod 5 propodus length 1.7× height, longer than dactylus (Fig. 3A).

Male thoracic sternum generally smooth, pitted, setae at margins and sparsely on sternites; sternites 1–4 width ~1.9× length; sternites 1, 2 fused to form broad triangle; fused sternites 1, 2 demarcated from sternite 3 by sinuous transverse sulcus, sulcus lined with setae; sternites 3, 4 fused without obvious demarcation (Fig. 3C). Male sterno-pleonal cavity reaching anteriorly slightly beyond mid-length of cheliped coxa (Fig. 3C). Male pleonal locking tubercle positioned slightly posterior to mid-length of sternite 5 (Fig. 3E).

Male pleon large, sublinguiform; somites 3–6 progressively narrower; somite 6 width approximately 2.5× length; telson width 1.7× length; lateral margins almost straight, apex rounded (Fig. 3D).

G1 large, slightly sinuous, tip exceeding suture between thoracic sternites 4/5 in situ (Fig. 3E); subterminal segment length ~2.5× length of terminal segment (Fig. 4C). Subterminal segment outer margin slightly concave, outer distal margin bulging; terminal segment curved inwards, tip pointed upwards in dissected view, connected to a large meso-anterior-facing horseshoe shaped structure, large thick proximal pad on dorsal side (Fig. 4C, F, G). G2 subterminal segment tapering, slightly bent outwards distally, flagelliform terminal segment thick, ~1.6× length of subterminal segment, apex blunt (Fig. 4B).

#### Colour in life.

Generally drab camouflage-brown all over (Fig. 13A).

#### Habitat.

Typical habitat unknown. The only specimen collected was found at night on the side of a dirt road near the summit at approximately 1600 m above sea level. Multiple subsequent attempts to find this species in the same area failed to locate any more specimens with only *Megapleonumwangjiani* sp. nov. and *Eurusamonguangdongense* being found. The true habitat of this species remains elusive.

#### Distribution.

Dawuling Nature Reserve, Maoming City, Guangdong Province, China.

#### Etymology.

The species name is the Latin word *ferrumequinum*, meaning horseshoe. It alludes to the horseshoe-shaped structure on the G1 terminal segment of this species. Used as a noun in apposition.

#### Remarks.

*Megapleonumferrumequinum* sp. nov. is unique in its genus by its sublinguiform male abdomen (Fig. 3D, vs broadly triangular in all other congeners) and its peculiar G1, which is only slightly sinuous (Fig. 4C, vs more strongly sinuous in all other congeners) with a horseshoe-shaped structure in the terminal segment (Fig. 4F–H, vs absent in all other congeners). In possessing dense setation on the carapace margins and ambulatory legs, *M.ferrumequinum* sp. nov. is most similar to *M.falx* sp. nov. (see remarks for *M.falx* sp. nov.). More detailed comparisons can be found in Table 1.

### ﻿ Megapleonum wangjiani sp. nov.

Taxon classificationAnimaliaDecapodaPotamidae

C80D0340-D964-5FCD-8913-422A01FB4647

https://zoobank.org/8E66307C-9F4C-4466-85D1-C7CBE7D6DB52

**Figure 5.**
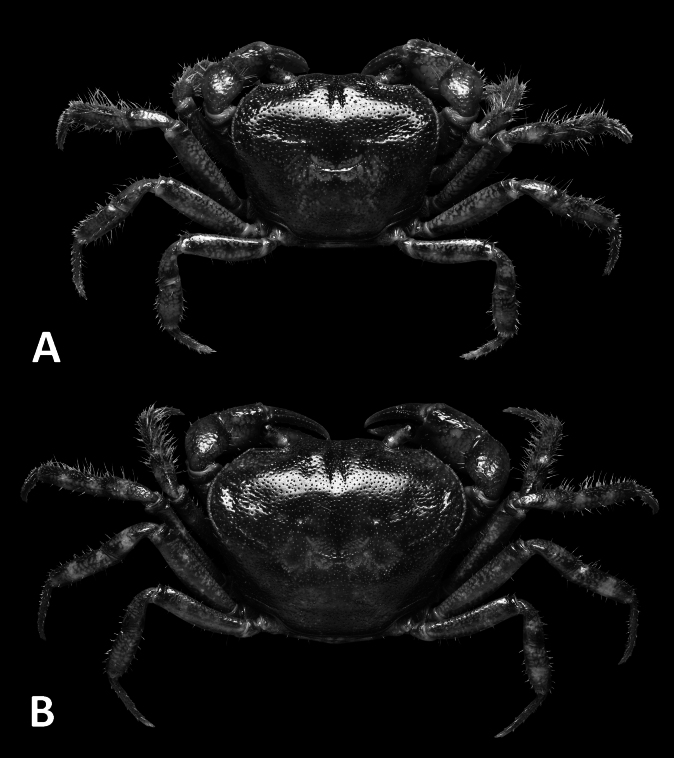
Dorsal habitus. *Megapleonumwangjiani* sp. nov., male holotype (11.7 × 9.7 mm), SYSBM 002144 (**A**); female paratype (16.7 × 12.8 mm), SYSBM 002148 (**B**).

**Figure 6.**
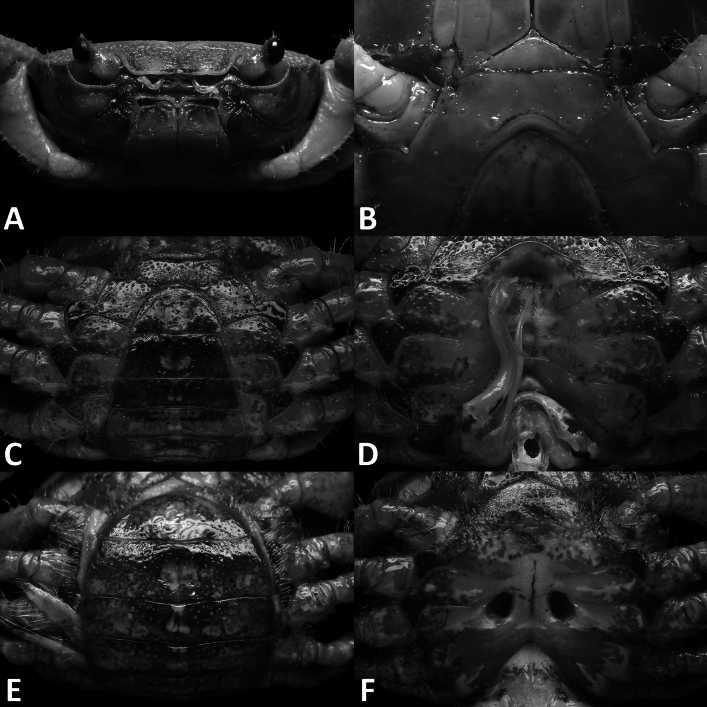
*Megapleonumwangjiani* sp. nov., male holotype (11.7 × 9.7 mm), SYSBM 002144 (**A**–**D**); female paratype (16.7 × 12.8 mm), SYSBM 002148 (**E**, **F**). Cephalothorax, anterior view (**A**); anterior thoracic sternum (**B**); anterior thoracic sternum and pleon, ventral view (**C**); sterno-pleonal cavity with G1 in situ, ventral view (**D**); pleon, ventral view (**E**); vulvae, ventral view (**F**).

**Figure 7.**
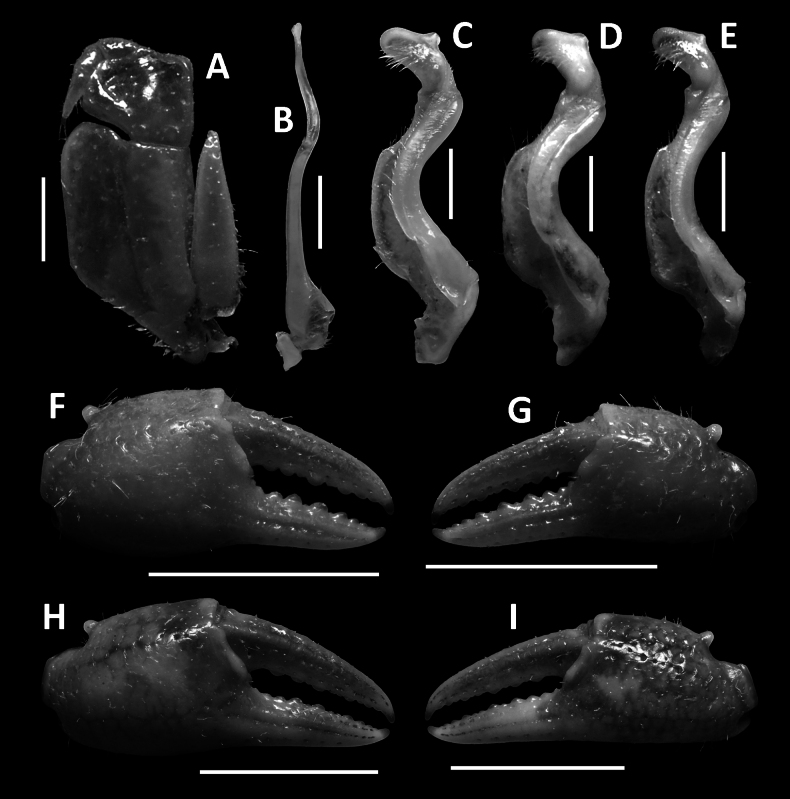
*Megapleonumwangjiani* sp. nov., male holotype (11.7 × 9.7 mm), SYSBM 002144 (**A**–**C**, **F**, **G**); male paratype (11.6 × 9.5 mm), SYSBM 002145 (**D**); male paratype (10.9 × 9.0 mm), SYSBM 002146 (**E**); female paratype (16.7 × 12.8 mm), SYSBM 00 2148 (**H**, **I**). Left maxilliped 3 (**A**); left G2, pleonal view (**B**); left G1, ventral view (**C**–**E**); major cheliped (**F**, **H**); minor cheliped (**G**, **I**). Scale bars: 1.0 mm **(A**–**E)**, 5.0 mm **(F**–**I)**.

Figs 5 , 6 , 7 , 11A, B , 13B, C

#### Type material.

***Holotype***: • SYSBM 002144, male (11.7 × 9.7 mm), Datianding, Dawuling Nature Reserve, Maoming City, Guangdong Province, China, 22.29°N, 111.22°E, under rocks in small seepage, coll. Chao Huang, November 2018. ***Paratypes***: • SYSBM 002145–002147, 3 males (11.6 × 9.5 mm, 10.9 × 9.0 mm, 8.3 × 6.9 mm), same data as holotype. SYSBM 002148–002151, 4 females (16.7 × 12.8 mm, 15.0 × 11.3 mm, 14.6 × 11.4 mm, 9.3 × 7.5 mm), same data as holotype. • NNU 16C-201811MW, 1 male (10.3 × 8.3 mm), 1 female (14.6 × 11.4 mm), same data as holotype.

#### Diagnosis.

Carapace broader than long; dorsal surface slightly convex, postorbital, epigastric cristae weak, rugose, almost confluent (Fig. 5). Maxilliped 3 merus width ~1.2× length; ischium width ~0.7× length; exopod reaching slightly beyond anterior edge of ischium, without flagellum (Fig. 7A). Ambulatory legs with dense setae; pereiopod dactylus shorter than propodus (Fig. 7). Male anterior thoracic sternum very broad, width ~2.0× length (Fig. 6B). Male pleon large, broadly triangular, pleonite 6 width ~2.6× length; telson width ~1.8× length (Fig. 6C). Female pleon subovate (Fig. 6E). G1 large, strongly sinuous, tip exceeding suture between thoracic sternites 4/5 in situ (Fig. 6D); subterminal segment length ~2.3× length of terminal segment (Fig. 7C–E). Subterminal segment outer margin strongly concave; terminal segment short, rounded, distoventrally with long setae, directed inwards, inner-proximal margin concave; tip presenting as protrusion on higher two-thirds of outer margin (Figs 7C–E, 11A, B). G2 subterminal segment slightly bent outwards distally, flagelliform terminal segment thick, ~1.7× length of subterminal segment, apex blunt, slightly swollen (Fig. 7B).

#### Description.

Carapace broader than long, ~1.2× as wide as long in males (*n* = 4), ~1.3× as wide in mature females (*n* = 4); regions not pronounced, dorsal surface convex; surface finely pitted, anterolateral regions slightly rugose (Fig. 5). Frontal margin slightly sinuous, deflexed (Fig. 5). Epigastric cristae and postorbital cristae rugose, low, almost confluent; bifurcated shallow groove between epigastric cristae (Fig. 5). Branchial regions not swollen (Fig. 5). Cervical groove shallow (Fig. 5). Mesogastric region flat (Fig. 5). External orbital angle broadly triangular, outer margin slightly convex, confluent with anterolateral margin (Figs 5, 6A). Epibranchial tooth granular, indistinct (Figs 5, 6A). Anterolateral margin lined with 10–14 granules; posterolateral margin posteriorly convergent (Fig. 5); posterolateral surface smooth (Fig. 5). Orbits regular; supraorbital margins weakly cristate, infraorbital margins lined with fused granules (Fig. 6A). Eyes normal (Figs 5, 6A). Sub-orbital, pterygostomial and sub-hepatic regions generally smooth, pitted (Fig. 6A). Antennules large, folded within broad fossae; antennae very short (Fig. 6A). Median lobe of epistome buccal margin triangular, lateral margins straight (Fig. 6A).

Maxilliped 3 merus subtrapezoidal, with slight median depression, width ~1.2× length; ischium subtrapezoidal with shallow median sulcus, distomesial margin rounded, width ~0.7× length. Exopod reaching proximal one-third of merus; flagellum absent (Fig. 4A).

Chelipeds (pereiopod 1) subequal (Fig. 7F–I). Merus trigonal in cross section, surfaces generally smooth; outer dorsal margin slightly crenulated, inner and ventral margin lined with large granules (Figs 5, 6A). Carpus dorsal surface slightly rugose, with small blunt spine at inner-distal angle, spinule at base (Fig. 5). Major cheliped palm length ~1.3–1.4× height in males (*n* = 2), 1.4–1.5× height in females (*n* = 4); dactylus 0.9× palm length in males (*n* = 2), 0.9–1.0× palm length in females (*n* = 4) (Fig. 7F–I). Palm surface pitted, occlusal margin of fingers with 8–11 irregular blunt teeth, with very small gape when closed (Fig. 7F–I).

Ambulatory legs slender (pereiopods 2–5) covered with setae, especially dense on pereiopods 2, 3 (Fig. 5). Pereiopod 3 merus 0.6–0.7× CL in both sexes (*n* = 8, Fig. 5). Pereiopod 5 propodus length 1.9–2.2× height in males (*n* = 4), 2.1–2.4× height in females (*n* = 4), shorter than dactylus (Fig. 5).

Male thoracic sternum generally smooth, sparsely pitted; sternites 1–4 width ~2.0× length; sternites 1, 2 fused to form broad triangle; fused sternites 1, 2 demarcated from sternite 3 by almost straight transverse sulcus; sternites 3, 4 fused without obvious demarcation (Fig. 6B). Male sterno-pleonal cavity reaching anteriorly slightly beyond mid-length of cheliped coxa (Fig. 6B). Male pleonal locking tubercle positioned at mid-length of sternite 5 (Fig. 6D). Female vulvae ovate, large, reaching suture of sternites 5/6, relatively widely separated (Fig. 6F).

Male pleon large, broadly triangular; somites 3–6 progressively narrower; somite 6 width ~2.6× length; telson width 1.8× length; lateral margins almost straight, apex rounded (Fig. 6C). Female pleon sub-ovate (Fig. 6E).

G1 large, strongly sinuous, tip exceeding suture between thoracic sternites 4/5 in situ (Fig. 6D); subterminal segment length ~2.3× length of terminal segment (Fig. 7C–E). Subterminal segment outer margin strongly concave; terminal segment short, rounded, distoventral region with long setae, directed inwards, inner-proximal margin concave; tip presenting as protrusion on higher two-thirds of outer margin (Figs 7C–E, 11A, B). G2 subterminal segment slightly bent outwards distally, flagelliform terminal segment thick, ~1.7× length of subterminal segment, apex blunt, slightly swollen (Fig. 7B).

#### Colour in life.

Generally drab camouflage-brown all over, some individuals exhibit a reddish hue (Fig. 13B, C).

#### Habitat.

Little is known about the ecology of this new species, aside from its occurrence at high elevations, where it inhabits seepages and is occasionally seen roaming the forest floor. *Megapleonumferrumequinum* sp. nov. and *Eurusamonguangdongense* are also found on the same mountain, but the three species apparently occupy different niches.

#### Distribution.

Dawuling Nature Reserve, Maoming City, Guangdong Province, China.

#### Etymology.

This species is named in honour of its discoverer, Jian Wang, in recognition of his contribution to this study.

#### Remarks.

Like many species in this genus, *Megapleonumwangjiani* sp. nov. can immediately be distinguished by its distinctive G1, especially in the rounded terminal segment with the tip presenting as a protrusion on the higher two-thirds of the outer margin (Figs 7C–E, 11A, B). Apart from the G1, *M.wangjiani* can be separated from the sympatric *M.ferrumequinum* sp. nov. by its slenderer legs, with the pereiopod 5 propodus length 1.9–2.4× height, shorter than dactylus, whereas in the latter the pereiopod 5 propodus length is 1.7× height, longer than dactylus (Fig. 5A, B vs Fig. 3A). *Megapleonumwangjiani* sp. nov. also has a broader male abdomen than *M.ferrumequinum* n. sp. (Fig. 6C vs Fig. 3D). More detailed comparisons can be found in Table 1.

There is noticeable sexual dimorphism in *Megapleonumwangjiani* sp. nov., with males being smaller and maturing at a smaller size than females (the male SYSBM 002147 at CW 8.3 mm has a full length G1 whereas the female SYSBM 002151 at CW 9.3 mm has an immature narrow abdomen). Females also have a proportionally wider carapace (1.2× as wide as long in mature males vs ~1.3× in mature females) and more slender legs than females (pereiopod 5 propodus length 1.9–2.2× height in males vs 2.1–2.4× height in females), but these differences might also be related to size and are only obvious due to the apparent inability for the males to reach the size of females. Interspecific variation of the G1 is small, with the tip opening varying slightly in size and shape (Fig. 7C–E).

### ﻿ Megapleonum yangdongense sp. nov.

Taxon classificationAnimaliaDecapodaPotamidae

9E1C850A-B36F-51BD-9223-39DC594E89F8

https://zoobank.org/63BEBAEA-6C90-4FCE-809D-7E4C7FBD62FF

**Figure 8.**
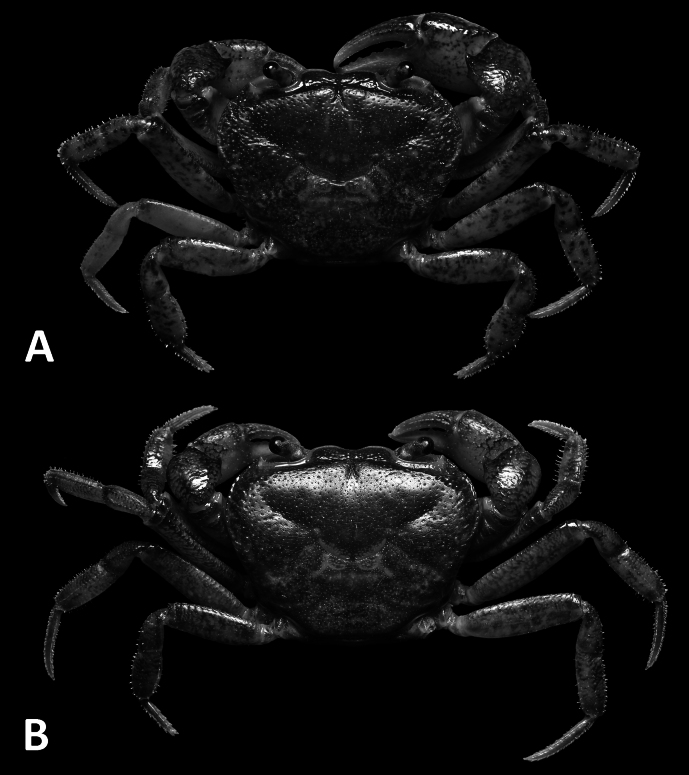
Dorsal habitus. *Megapleonumyangdongense* sp. nov., male holotype (17.8 × 14.5 mm), SYSBM 002152 (**A**); female paratype (16.0 × 12.4 mm), SYSBM 002154 (**B**).

**Figure 9.**
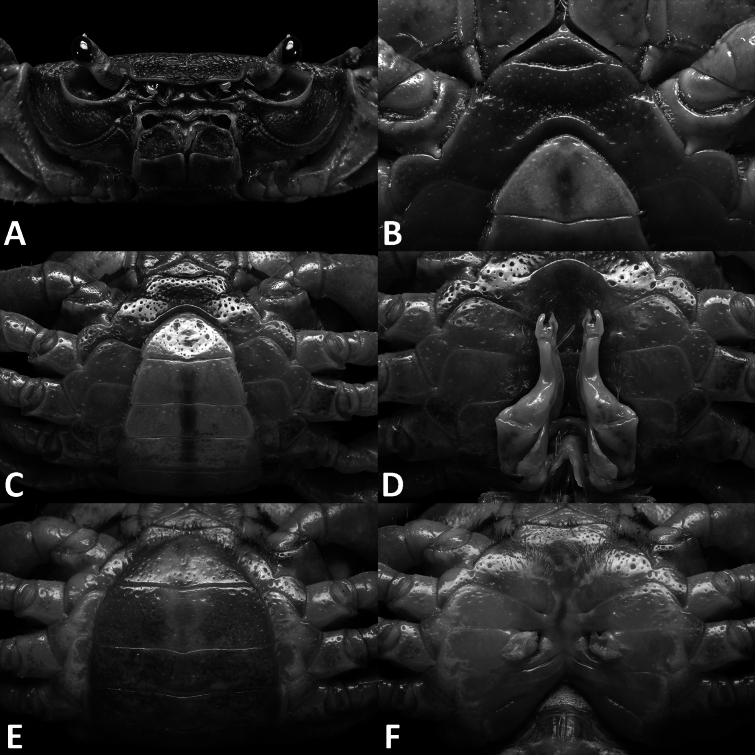
*Megapleonumyangdongense* sp. nov., male holotype (17.8 × 14.5 mm), SYSBM 002152 (**A**–**D**); female paratype (16.0 × 12.4 mm), SYSBM 002154 (**E**, **F**). Cephalothorax, anterior view (**A**); anterior thoracic sternum (**B**); anterior thoracic sternum and pleon, ventral view (**C**); sterno-pleonal cavity with G1 in situ, ventral view (**D**); pleon, ventral view (**E**); vulvae, ventral view (**F**).

**Figure 10.**
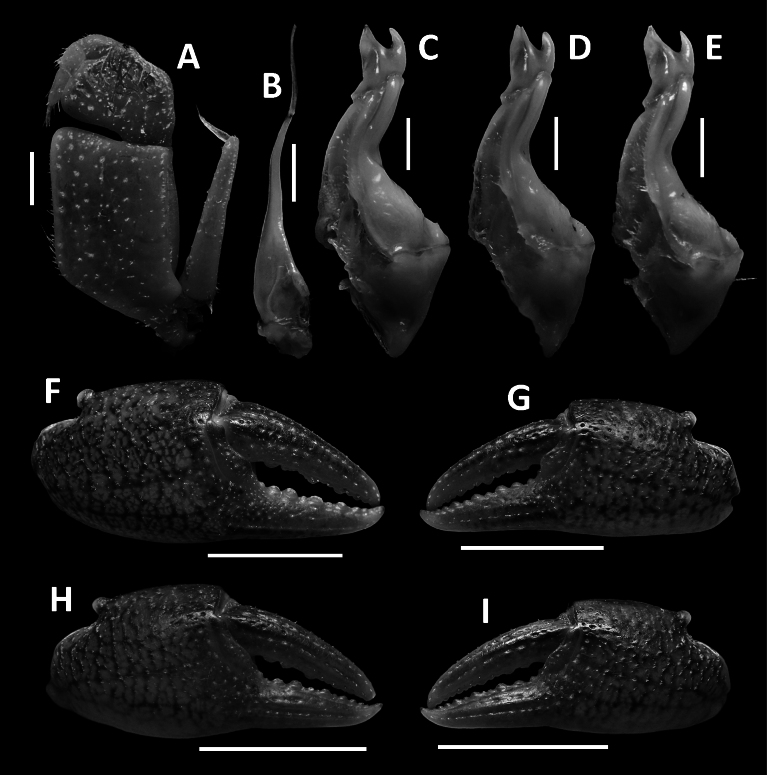
*Megapleonumyangdongense* sp. nov., male holotype (17.8 × 14.5 mm), SYSBM 002152 (**A**–**C**, **F**, **G**); male paratype (11.6 × 9.5 mm), SYSBM 002153 (**D**); male paratype (16.0× 12.7 mm), IHB (**E**); female paratype (16.0 × 12.4 mm), SYSBM 002154 (**H**, **I**). Left maxilliped 3 (**A**); left G2, pleonal view (**B**); left G1, ventral view (**C**–**E**); major cheliped (**F**, **H**); minor cheliped (**G**, **I**). Scale bars: 1.0 mm **(A**–**E)**, 5.0 mm **(F**–**I)**.

**Figure 11.**
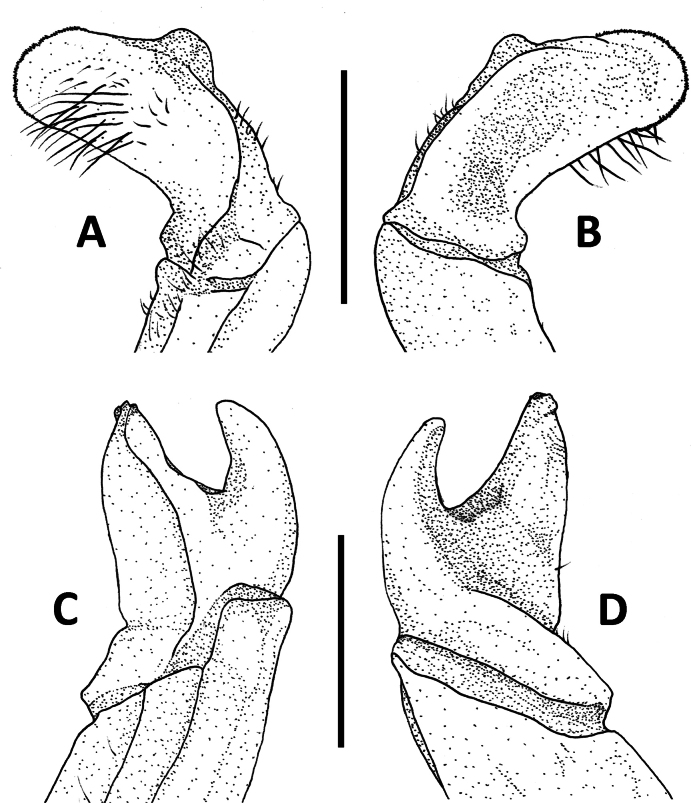
Left G1 terminal segments. *Megapleonumwangjiani* sp. nov., male holotype (11.7 × 9.7 mm), SYSBM 002144 (**A**, **B**); *Megapleonumyangdongense* sp. nov., male holotype (17.8 × 14.5 mm), SYSBM 002152(**C**, **D**). Ventral view (**A**, **C**); dorsal view (**B**, **D**). Scale bar: 1.0 mm.

Figs 8 , 9 , 10 , 11C, D , 13D

#### Type material.

***Holotype***: • SYSBM 002152, male (17.8 × 14.5 mm), Gaozhai, Yangdong County, Yangjiang City, Guangdong Province, China, 21.97°N; 112.07°E, under rocks in hillstream, coll. Chao Huang, Hsi-Te Shih and Bernhard Bein, 1 June 2019. ***Paratypes***: • SYSBM 002153, male (17.3 × 13.7 mm), same data as holotype. SYSBM 002154–002155, 2 females (16.0 × 12.4 mm, 15.3 × 12.0 mm), same data as holotype. IHB, 2 males (16.0 × 12.7 mm, 15.4 × 12.0 mm), same data as holotype. NCHUZOOL 15306, 2 males (16.0 × 13.1 mm, 14.9 × 11.8 mm), 1 female (16.0 × 12.6 mm), NCHUZOOL 15307, 1 male (16.7 × 13.8 mm), same data as holotype.

#### Diagnosis.

Carapace broader than long, dorsal surface slightly convex, postorbital, epigastric cristae confluent, sharp (Fig. 8). Maxilliped 3 merus width ~1.3× length; ischium width ~0.7× length; exopod reaching slightly beyond anterior edge of ischium; flagellum short, as long as dactylus (Fig. 10A). Ambulatory legs without long setae, pereiopod dactylus shorter than propodus (Fig. 8). Male anterior thoracic sternum very broad, width ~1.9× length (Fig. 9B). Male pleon large, broadly triangular, pleonite 6 width ~2.4× length, telson width ~1.8× length (Fig. 9C). Female pleon subovate (Fig. 9E). G1 large, sinuous, tip exceeding suture between thoracic sternites 4/5 in situ (Fig. 9D); subterminal segment length ~2.7× length of terminal segment (Fig. 10C–E); subterminal segment thick, outer margin strongly concave, distal end slanted with outer-distal section highest; terminal segment short, both lateral margins slightly convex, bifurcated with one point being opening tip and other a smaller projection on outer margin, both pointing upwards (Figs 10C–E, 11C, D). G2 subterminal segment thick, slightly bent outwards distally, flagelliform terminal segment thin, ~2.3× length of subterminal segment (Fig. 10B).

#### Description.

Carapace broader than long, males ~1.2–1.3× as wide as long (*n* = 6); regions not all visible, dorsal surface slightly convex; surface pitted, anterolateral regions slightly rugose (Fig. 8). Frontal margin sinuous, deflexed (Fig. 8). Epigastric cristae and postorbital cristae confluent, sharp; bifurcated shallow groove between epigastric cristae (Fig. 8). Branchial regions not swollen (Fig. 8). Cervical groove obvious (Fig. 8). Mesogastric region flat (Fig. 8). External orbital angle broadly triangular, outer margin slightly convex, almost confluent with anterolateral margin (Figs 8, 9A). Epibranchial tooth granular, indistinct (Figs 8, 9A). Anterolateral margin lined with 10–14 granules; posterolateral margin posteriorly convergent (Fig. 8); posterolateral with weak striae (Fig. 8). Orbits regular; supraorbital margins cristate, infraorbital margins lined with fused granules (Fig. 9A). Eyes normal (Figs 8, 9A). Sub-orbital, pterygostomial and sub-hepatic regions generally smooth, pitted (Fig. 9A). Antennules large, folded within broad fossae; antennae very short (Fig. 9A). Median lobe of epistome buccal margin broadly triangular, lateral margins straight (Fig. 9A).

Maxilliped 3 merus subtrapezoidal, with slight median depression, width ~1.3× length; ischium subtrapezoidal with very shallow median sulcus, distomesial margin rounded, width ~0.7× length. Exopod reaching proximal one-third of merus; flagellum short (Fig. 10A).

Chelipeds (pereiopod 1) subequal (Fig. 10F–I). Merus trigonal in cross section, surfaces generally smooth; outer dorsal margin slightly crenulated, inner and ventral margin lined with large granules (Figs 8, 9A). Carpus dorsal surface slightly rugose, with small blunt spine at inner-distal angle, spinule at base (Fig. 8). Major cheliped palm length ~1.4–1.5× height in males (*n* = 3), 1.4× height in females (*n* = 2); dactylus 0.9× palm length in males (*n* = 3), 0.8–0.9× palm length in females (*n* = 2) (Fig. 10F–I). Palm surface pitted, occlusal margin of fingers with 9–13 irregular blunt teeth, with very small gape when closed (Fig. 10F–I).

Ambulatory legs slender (pereiopods 2–5), with only very short setae on margins (Fig. 8). Pereiopod 3 merus 0.7× CL in both sexes (*n* = 5, Fig. 8). Pereiopod 5 propodus length 1.7–2.0× height in males (*n* = 3), 1.8–1.9× height in females (*n* = 2), longer than dactylus (Fig. 8).

Male thoracic sternum generally smooth, sparsely pitted; sternites 1–4 width ~1.9× length; sternites 1, 2 fused to form broad triangle; fused sternites 1, 2 demarcated from sternite 3 by slightly sinuous sulcus; sternites 3, 4 fused with distinct sulcus (Fig. 9B). Male sterno-pleonal cavity reaching anteriorly slightly beyond mid-length of cheliped coxa (Fig. 9B). Male pleonal locking tubercle positioned at mid-length of sternite 5 (Fig. 9D). Female vulvae ovate, large, reaching suture of sternites 5/6, relatively widely separated (Fig. 9F).

Male pleon large, broadly triangular; somites 3–6 progressively narrower; somite 6 width approximately 2.4× length; telson width 1.8× length; lateral margins almost straight, apex rounded (Fig. 6C). Female pleon sub-ovate (Fig. 9E).

G1 large, sinuous, tip exceeding suture between thoracic sternites 4/5 in situ (Fig. 9D); subterminal segment length ~2.7× length of terminal segment (Fig. 10C–E). Subterminal segment thick, outer margin strongly concave, distal end slanted with outer-distal section highest; terminal segment short, both lateral margins slightly convex, bifurcated with one point being opening tip and other being smaller projection on outer margin, both pointing upwards (Figs 10C–E, 11C, D). G2 subterminal segment thick, slightly bent outwards distally, flagelliform terminal segment thin, ~2.3× length of subterminal segment (Fig. 10B).

#### Colour in life.

Generally camouflaged in light mottled brown overall (Fig. 13D).

#### Habitat.

This is a typical aquatic hill stream species that can be found residing under rocks in the shallows of the hillstream. The sympatric *Eurusamonguangdongense* is also aquatic but can grow to a much larger size and mature individuals occupy the deeper areas of the hillstream.

#### Distribution.

Yangdong County, Yangjiang City, Guangdong Province, China.

#### Etymology.

This species is named after the type locality, Yangdong County.

#### Remarks.

This new species is closest to *Megapleonumehuangzhang* and especially *M.shenzhen* in terms of carapace and gonopodal morphology, and the three are no doubt closely related. *Megapleonumyangdongense* sp. nov., however, can be distinguished from the other two by its prong-shaped G1 terminal segment (vs folded terminal segment in *M.ehuangzhang*, Huang et al. 2018: fig. 3C, and goose-head-shaped terminal segment in *M.shenzhen*, Huang and Mao 2021: fig. 3C–E). Otherwise, it can further be separated from the two aforementioned congeners in having a slightly narrower male abdominal somite 6, with the width ~2.4× length (vs 2.6× in both *M.ehuangzhang* and *M.shenzhen*, Huang et al. 2018; Huang and Mao 2021). More detailed comparisons can be found in Table 1. Interspecific variation of the G1 is minimal, with the terminal segment slightly varying in size and the outer projection of the terminal segment slightly varying in sharpness and angle (Fig. 10C–E).

## ﻿DNA analyses and discussion

**Figure 12.**
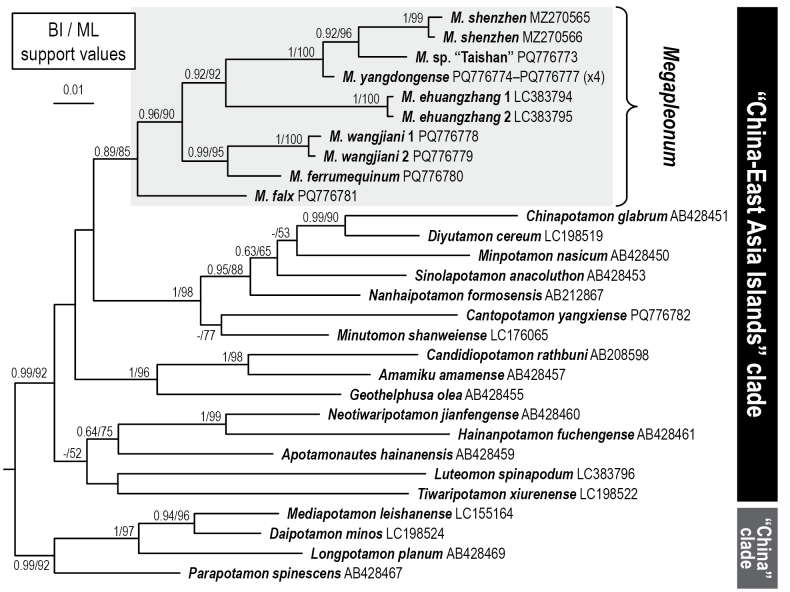
A Bayesian inference (BI) tree of 16S rDNA for *Megapleonum* and related taxa. Support values at nodes represent posterior probabilities and bootstrap proportions > 50% for BI and maximum likelihood (ML), respectively.

A 503 bp segment of the 16S rDNA, excluding variable regions, was amplified and aligned. The accession numbers for the 16S sequences of the new species of *Megapleonum* are as follows: *M.falx* sp. nov. (PQ776781), *M.ferrumequinum* sp. nov. (PQ776780), *M.wangjiani* sp. nov. (PQ776778, PQ776779), and *M.yangdongense* sp. nov. (PQ776774–PQ776777). BI and ML analyses based on the 16S sequences produced similar phylogenetic topologies (Fig. 12). The genus, which includes seven species, is monophyletic and deep-rooted, with *M.falx* sp. nov. occupying the basal position as sister to a lineage comprising two major clades. One of the major clades contains the lowland coastal species *M.ehuangzhang*, *M.shenzhen*, *M.* sp. “Taishan” and *M.yangdongense* sp. nov., whereas the other major clade includes two sympatric species from highland inland habitat, *M.ferrumequinum* sp. nov. and *M.wangjiani* sp. nov.

**Figure 13.**
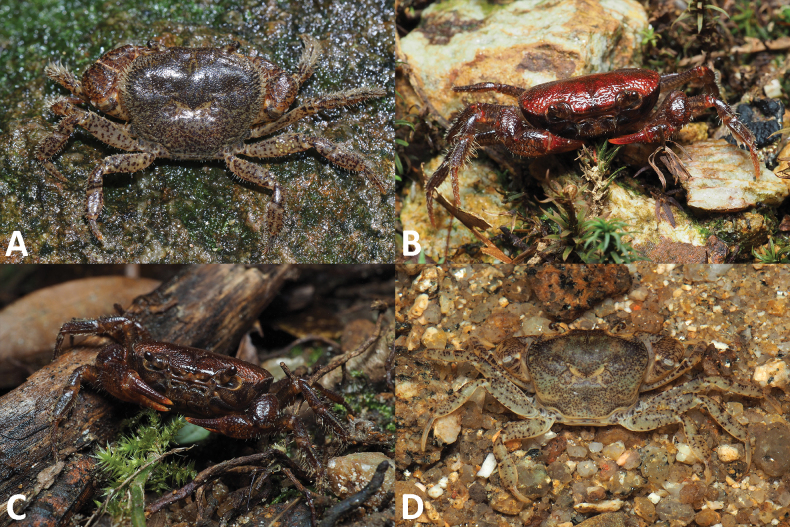
Colour in life. *Megapleonumferrumequinum* sp. nov., male (**A**); *Megapleonumwangjiani* sp. nov., male (**B**); *Megapleonumwangjiani* sp. nov., female (**C**); *Megapleonumyangdongense* sp. nov., male.

The phylogenetic relationships among species in this genus align with their distributions, although the basal species, *M.falx*, with a long maxilliped 3 exopod flagellum, occurs further northeast than its congeners. Notably, the long maxilliped 3 exopod flagellum in *M.falx* reflects the plesiomorphic condition, which is consistent with its basal position in the genus. The remaining species are split into two major clades: the lowland coastal clade and the highland inland clade. The lowland coastal clade has a uniform west to east distribution pattern that suggests historical eastward expansion or divergence. The westernmost species (*M.ehuangzhang* from Yangjiang) is sister to a clade that has *M.yangdongense* n. sp. (distributed east of *M.ehuangzhang* but also from Yangjiang) as the sister to *M.shenzhen* + *M.* sp. “Taishan” from further east. The species in this clade have a similar carapace physiognomy, short to absent maxilliped 3 exopod flagellum, comparable G1, especially *M.yangdongense* sp. nov., *M.shenzhen*, *M.* sp. “Taishan”, and do not possess long dense setae. The two sympatric species from the highland inland clade, *M.ferrumequinum* sp. nov. and *M.wangjiani* sp. nov. from Maoming, occur at the westernmost boundary of the genus and are both further from the coast than all the others. Both these species lack the maxilliped 3 exopod flagellum and possess dense long setae, especially on the pereiopods 2 and 3, and also on the carapace lateral margins in the former. Possessing long dense setae is rare among Chinese potamids and is observed only in *M.falx* sp. nov., *Barbamonzhoui* Shi, Pan & Sun, 2022, *Minutomonshanweiense* Huang, Mao & Huang, 2014, and some species of the genus *Pusillamon* Shi, Yeo & Sun, 2023. Despite sharing this character, these crabs differ in their habitat and ecology, which suggests that long dense setae may serve different functions in different situations, such as facilitating water uptake in semiterrestrial crabs or enhancing sensory abilities in aquatic crabs (Kawamura et al. 2021; Watson-Zink 2021). The specific functions of setae in these crabs will have to be determined by future ecological studies. However, this setal characteristic is likely to be a derived trait, as these genera are not closely related (Fig. 12; unpublished data).

The pairwise nucleotide divergences and the number of bp differences in the 556-bp 16S region within and between species are shown in Table 2, with values from 2.07% and 11 bp to 8.88% and 45 bp, respectively. Although the minimum interspecific divergence values in this genus are smaller than those of some other species, e.g. *Cantopotamon* (4.63% between *C.hengqinense* Huang, Ahyong & Shih, 2017 and *C.zhuhaiense* Huang, Ahyong & Shih, 2017, recalculated from Huang et al. 2017) and *Candidiopotamon* (3.22% between *C.rathbuni* (De Man, 1914) and *C.penglai* Shih, Naruse & Schubart, 2023, recalculated from Shih et al. 2006, 2023c), the values are larger than those of others, e.g., *Minpotamon* (1.47% between *M.nasicum* (Dai, Chen, Song, Fan, Lin & Zeng, 1979) and *M.kityang* Mao & Huang, 2020, Mao and Huang 2020) and *Nanhaipotamon* (≤ 1.12% among six species of the “Strait clade”, recalculated from Shih et al. 2011). As such, the four new *Megapleonum* species are genetically supported by 16S rDNA (Table 2). In crab taxonomy, the 16S marker has been commonly used for species delimitation (e.g., Shih et al. 2004, 2005, 2008; Huang 2018; Schubart and Ng 2020). Recently, however, the cytochrome c oxidase subunit I (COI) barcoding marker, with ~2.5× the divergence rate of 16S (Schubart et al. 1998; Shih et al. 2007), has been widely adopted (see Chu et al. 2015; Shih et al. 2022b, c). This marker can detect closely related or pseudocryptic species (e.g., Shy et al. 2014; Hsu et al. 2022; Prema et al. 2022; Shih et al. 2023b) as well as intraspecific differentiation (e.g., Shih et al. 2022a, 2023a). It is expected that further extensive surveys will reveal additional species of *Megapleonum*, detectable through the COI barcoding marker.

**Table 2.** Matrix of percent pairwise nucleotide divergence and the number of bp differences among the species of *Megapleonum* based on the 16S rDNA gene. In the right half, lower-left values are K2P distance and upper-right ones are bp differences. Values of the range are given in parentheses.

	Within species		Between species						
	Nucleotide divergence	bp difference	*M.shenzhen*	*M.* sp. “Taishan”	*M.yangdongense* sp. nov.	*M.ehuangzhang*	*M.wangjiani* sp. nov.	*M.ferrumequinum* sp. nov.	*M.falx* sp. nov.
*M.shenzhen*	0.18	1		14 (13–15)	11.5 (11–12)	41 (40–42)	38 (36–40)	34 (33–35)	34.5 (33–36)
*M.* sp. “Taishan”	0	0	2.64 (2.45–2.83)		11	38	39.5 (39–40)	37	37
*M.yangdongense* sp. nov.	0	0	2.16 (2.07–2.25)	2.07		33	30.5 (30–31)	31	33
*M.ehuangzhang*	0	0	8.01 (7.82–8.2)	7.35	6.35		37.5 (37–38)	37	45
*M.wangjiani* sp. nov.	0.18	1	7.4 (7.01–7.8)	7.68 (7.57–7.78)	5.85 (5.75–5.96)	7.25 (7.14–7.35)		15.5 (15–16)	33.5 (33–34)
*M.ferrumequinum* sp. nov.	0	0	6.57 (6.39–6.76)	7.16	5.96	7.17	2.9 (2.81–3)		32
*M.falx* sp. nov.	0	0	6.69 (6.4–6.99)	7.19	6.38	8.88	6.48 (6.37–6.58)	6.17	

## Supplementary Material

XML Treatment for Megapleonum

XML Treatment for Megapleonum falx

XML Treatment for Megapleonum ferrumequinum

XML Treatment for Megapleonum wangjiani

XML Treatment for Megapleonum yangdongense

## ﻿Additional information

### Author ORCIDs

Chao Huang https://orcid.org/0000-0002-3782-9096

Hsi-Te Shih https://orcid.org/0000-0003-1317-8783

Shane T. Ahyong https://orcid.org/0000-0002-2820-4158

### Data availability

All of the data that support the findings of this study are available in the main text.
